# Solid-State-Activated Sintering of ZnAl_2_O_4_ Ceramics Containing Cu_3_Nb_2_O_8_ with Superior Dielectric and Thermal Properties

**DOI:** 10.3390/ma15051770

**Published:** 2022-02-26

**Authors:** Koichi Shigeno, Takuma Yano, Hirotaka Fujimori

**Affiliations:** 1 National Institute of Technology (KOSEN), Ube College, 2-14-1 Tokiwadai, Ube, Yamaguchi 755-8555, Japan; ntnm.894@gmail.com; 2 Graduate School of Sciences and Technology for Innovation, Yamaguchi University, 2-16-1 Tokiwadai, Ube, Yamaguchi 755-8611, Japan; hiro@hiro-fuji.net

**Keywords:** ZnAl_2_O_4_ (gahnite), low-temperature co-fired ceramics (LTCCs), solid-state-activated sintering, microwave dielectric properties, thermal conductivity

## Abstract

Low-temperature co-fired ceramics (LTCCs) are dielectric materials that can be co-fired with Ag or Cu; however, conventional LTCC materials are mostly poorly thermally conductive, which is problematic and requires improvement. We focused on ZnAl_2_O_4_ (gahnite) as a base material. With its high thermal conductivity (~59 W·m^−1^·K^−1^ reported for 0.83ZnAl_2_O_4_–0.17TiO_2_), ZnAl_2_O_4_ is potentially more thermally conductive than Al_2_O_3_ (alumina); however, it sinters densely at a moderate temperature (~1500 °C). The addition of only 4 wt.% of Cu_3_Nb_2_O_8_ significantly lowered the sintering temperature of ZnAl_2_O_4_ to 910 °C, which is lower than the melting point of silver (961 °C). The sample fired at 960 °C for 384 h exhibited a relative permittivity (*ε_r_*) of 9.2, a quality factor by resonant frequency (*Q* × *f*) value of 105,000 GHz, and a temperature coefficient of the resonant frequency (*τ_f_*) of −56 ppm·K^−1^. The sample exhibited a thermal conductivity of 10.1 W·m^−1^·K^−1^, which exceeds that of conventional LTCCs (~2–7 W·m^−1^·K^−1^); hence, it is a superior LTCC candidate. In addition, a mixed powder of the Cu_3_Nb_2_O_8_ additive and ZnAl_2_O_4_ has a melting temperature that is not significantly different from that (~970 °C) of the pristine Cu_3_Nb_2_O_8_ additive. The sample appears to densify in the solid state through a solid-state-activated sintering mechanism.

## 1. Introduction

Low-temperature co-fired ceramics (LTCCs) are dielectric materials that can be co-fired with Ag or Cu, which are metals that exhibit low-resistance conduction at temperatures below their melting points (961 and 1084 °C, respectively) [1,2]. LTCCs have been widely used in small electronic devices, such as wiring substrates and integrated circuit packages for high-frequency communication. With the aim of improving energy efficiency, ultralow-temperature co-fired ceramics (ULTCCs) that can be co-fired with Al at 660 °C (below the melting point of Al) are currently being actively researched [3]. However, the poor heat-dissipation properties of LTCC materials are problematic because the temperature of semiconductors mounted on the LTCC materials rises, causing thermal runaway of these semiconductors. Aluminum-based oxide ceramics, such as Al_2_O_3_ (alumina), are relatively highly thermally conductive; however, a large amount (approximately 50% or more of the total) of a poorly thermally conductive low-softening-point glass needs to be added to achieve low-temperature sintering when used as the base material. Consequently, the majority of these conventional LTCC materials are poorly thermally conductive (approximately 2–7 W·m^−1^·K^−1^), which is a shortcoming [4,5]. The heat-generation densities of semiconductor-based electronic components, such as light-emitting diodes (LEDs), mounted on LTCC multilayer devices have recently been reported to be increasing [6,7]; hence, highly thermally conductive LTCC materials are in demand. We previously developed sintering additives for alumina using highly thermally conductive (~30 W·m^−1^·K^−1^) alumina as a base material that, when added in small amounts, enables alumina to be sintered at low temperatures. As a result, we developed a CuO–TiO_2_–Nb_2_O_5_–Ag_2_O additive that facilitates alumina sintering at 900 °C or less when added at 5 wt.%, thereby realizing highly thermally conductive (~18–20 W·m^−1^·K^−1^) low-temperature co-fired alumina (LTCA) [8,9].

Furthermore, we also focused on ZnAl_2_O_4_ (gahnite) as a base material; this aluminum-based oxide has good dielectric properties and is potentially more thermally conductive than alumina [10,11,12,13,14,15,16]. According to Surendran et al., a sample produced by firing a 0.83ZnAl_2_O_4_–0.17TiO_2_ (molar ratio) composition at 1440 °C exhibited a thermal conductivity of 59 W·m^−1^·K^−1^ [11]. Consequently, ZnAl_2_O_4_ is expected to be used as a novel highly thermally conductive LTCC substrate if it can be sintered at low temperature. Low-temperature sintering involving the addition of glass to ZnAl_2_O_4_ [14] has been studied. The use of crystalline additives other than glass has also been studied a few times [16]; however, densely sintered ZnAl_2_O_4_ has not been achieved below 1100 °C yet. Therefore, we aimed to develop a sintering additive that, when added in small amounts, enables ZnAl_2_O_4_ to be densely sintered below 1000 °C (a low temperature).

We previously found that the addition of only 5 wt.% CuO–Nb_2_O_5_ (in a 7:3 Cu:Nb molar ratio) led to a significantly lower sintering temperature (960 °C) [17]. The sample fired at 960 °C for 2 h exhibited a relative permittivity (*ε_r_*) of 9.1, a quality factor by resonant frequency (*Q* × *f*) value of 30,000 GHz (at a frequency of ~13 GHz), a temperature coefficient of the resonant frequency (*τ_f_*) of −69 ppm·K^−1^, and a thermal conductivity (*κ*) of 9.3 W·m^−1^·K^−1^, which are relatively satisfactory; however, these values can be further improved. Because the mixture of CuO and Nb_2_O_5_ is separated at the atomic level, we postulate that small amounts of unreacted components exacerbate the *Q* × *f* value of low-temperature sintered ZnAl_2_O_4_. In addition, the low-temperature sintering mechanism remains unknown. The composite oxide formed between a ZnO varistor and an Al_2_O_3_ substrate by low-temperature sintering has also been recently examined [7]; ZnO containing Bi_2_O_3_ is known to react with Al_2_O_3_ to produce ZnAl_2_O_4_ [18]. Therefore, understanding the sintering behavior of ZnAl_2_O_4_ from a practical perspective is also important.

This study has two objectives. The first involves further improving the dielectric properties and thermal conductivities of low-temperature sintered ZnAl_2_O_4_ ceramics. To achieve this goal, we examined adding small quantities of calcined Cu_3_Nb_2_O_8_ [19], which has been reported to exhibit relatively good dielectric properties, instead of a mixture of CuO and Nb_2_O_5_ in the same molar ratio, prolonging the holding time at each firing temperature. The second involves gaining insight into the low-temperature sintering mechanism. Specifically, we examined whether densification is promoted only in the solid phase or in other phases, including the liquid phase. In addition, we examined the densification role of each additive component (Cu and Nb).

## 2. Materials and Methods

### 2.1. Fabricating Sintered Samples

**Figure 1:**
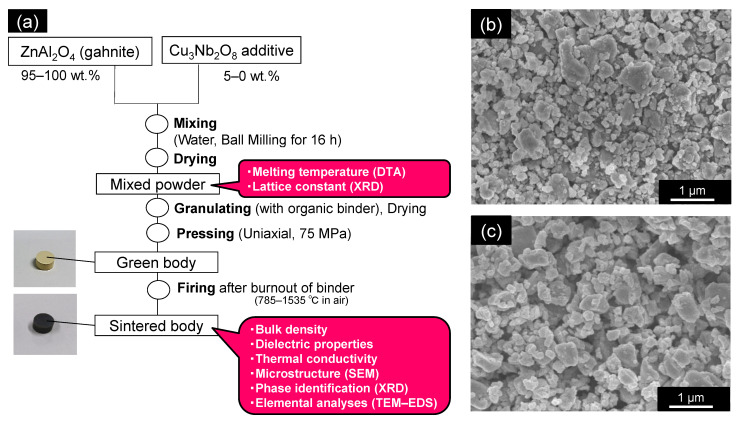
(**a**) Experimental process for preparing ZnAl_2_O_4_ ceramics containing the Cu_3_Nb_2_O_8_ sintering aid. (**b**) Scanning electron microscopy (SEM) image of the ZnAl_2_O_4_ raw material powder. (**c**) SEM image of the Cu_3_Nb_2_O_8_ raw material powder.

Figure 1a shows the flow process used to prepare gahnite sintered bodies containing additives. First, commercially available Al_2_O_3_ and ZnO powders (99.99%, average particle size: 1 μm for each powder; Kojundo Chemical Laboratory Co., Ltd., Saitama, Japan) were mixed in a 1:1 molar ratio and ball-milled for 16 h, with water as the dispersion medium. The dried powder was then calcined at 1100 °C for 4 h in air to yield ZnAl_2_O_4_, which was then pulverized using a ball mill for 48 h, with water as the dispersion medium. The dried powder was used as the raw material, as shown in Figure 1b; its specific surface area was determined to be 8.65 m^2^/g by the Brunauer–Emmett–Teller (BET) method (AUTOSORB-1, Spectris Co., Ltd., Kanagawa, Japan). According to the BET measurements and assuming particles were spherical [20], the average particle size was approximately 0.2 μm.

Table 1 lists the 14 main samples characterized in this study, as well as their synthesis conditions, and the purpose of each characteristic comparison. Commercially available CuO (99.3%, Nissin Chemco Ltd., Kyoto, Japan) and Nb_2_O_5_ (99.9%, Kojundo Chemical Laboratory Co., Ltd., Saitama, Japan) powders were mixed in a 3:2 Cu:Nb molar ratio and ball-milled for 16 h, with water as the dispersion medium. The dried powder was calcined at 835 °C for 2 h in air to yield Cu_3_Nb_2_O_8_, which was pulverized using a ball mill for 48 h, with water as the dispersion medium. The dried powder was used as the additive, as shown in Figure 1c. ZnAl_2_O_4_ powder (100–95 wt.%) and the Cu_3_Nb_2_O_8_ additive powder (0–5 wt.%) were mixed and ball-milled for 16 h, with water as the dispersion medium. We set the maximum additive concentration to 5 wt.% because we found that this quantity provided sufficiently well-sintered samples in our previous study [17]. For reference, ball-milling was conducted with yttria-stabilized zirconia (YSZ) balls in a polyethylene bottle at a rotation rate of 160 rpm.

The dried powder was granulated with polyvinyl alcohol (PVA, DKS Co., Ltd., Kyoto, Japan) binder and formed into a disc through uniaxial pressing at 75 MPa. The weight ratio of the dried powder–PVA binder was set to be 100:4. The green bodies were fired at 785–1535 °C in air for the required time (between 10 min and 384 h). The effects of sintering-aided calcination on sinterability and the dielectric and thermal properties were investigated. Specifically, ZnAl_2_O_4_ samples were fabricated by separately adding CuO and Nb_2_O_5_ (5 wt.% in total) in the same Cu:Nb molar ratio (3:2) as Cu_3_Nb_2_O_8_ (referred to as “non-calcined” in Table 1). For reference, we also prepared a sintered body calcined at 885 °C for 2 h using only the Cu_3_Nb_2_O_8_ additive powder. For reference, the heating and cooling rates used for calcination and firing were both set to 300 °C/h.

### 2.2. Characterization

The properties of the prepared sintered bodies were evaluated on the basis of their ceramic densities, microwave dielectric properties, and thermal conductivities. The ceramic densities were measured using the geometrical method. The theoretical density (*ρ*) of each sample was calculated using the following equation:

(1) $$\rho =\frac{W_{1}+W_{2}}{\left(\frac{W_{1}}{\rho_{1}}\right)+\left(\frac{W_{2}}{\rho_{2}}\right)},$$

where *W*_1_ and *W*_2_, are the weight percentages of ZnAl_2_O_4_ and Cu_3_Nb_2_O_8_, respectively, and *ρ*_1_ and *ρ*_2_ are the densities of ZnAl_2_O_4_ (4.606 g/cm^3^) and Cu_3_Nb_2_O_8_ (5.655 g/cm^3^), respectively [21]. Relative density was calculated by dividing the measured ceramic density by the theoretical density. Three major parameters that describe microwave dielectric properties, namely, the relative permittivity (*ε_r_*), the quality factor by resonant frequency (*Q* × *f*), and the temperature coefficient of the resonant frequency (*τ_f_*), were measured using the Hakki–Coleman method [22] with a network analyzer (8720ES, Agilent Technologies, Santa Clara, CA, USA). *τ_f_* values were calculated using the following equation:

(2) $$\tau_{f}=\frac{1}{f\left(T_{0}\right)}\cdot \frac{f\left(T_{1}\right)-f\left(T_{0}\right)}{T_{1}-T_{0}},$$

where *f(T*_0_*)* and *f(T*_1_*)* are the resonant frequencies at 20 and 80 °C, respectively. Thermal conductivities were measured using the xenon flash method (LFA447, Netzsch, Selb, Germany). To discuss sinterability, as well as the microwave dielectric properties and thermal conductivities of the developed materials, we observed their microstructures by scanning electron microscopy (SEM; JSM-7600F, JEOL Ltd., Tokyo, Japan) at an accelerating voltage of 5 kV. To prepare samples for SEM, the samples were polished with 0.5 μm diamond abrasives, followed by thermal etching for 1 h at a temperature 50 °C lower than each firing temperature. The average grain size (*D_g_*) was measured by the planimetric method [20,23] for at least 200 grains. In addition, we identified their crystalline phases via X-ray diffractometry (XRD; Ultima IV, Rigaku Co., Ltd., Tokyo, Japan) augmented with a Cu-Kα radiation source at 40 kV and 40 mA in the 2θ range of 20–60° with a step size of 0.02° and a rate of 4°·min^−1^.

To further discuss the sintering mechanism, the melting temperatures of the mixed powders were determined by differential thermal analysis (DTA; Thermo Plus Evo II, Shimadzu Co., Ltd., Kyoto, Japan). The lattice constants of ZnAl_2_O_4_ and Cu_3_Nb_2_O_8_ in the mixed powders were determined via a step scanning method of XRD augmented with a Cu-Kα radiation source at 40 kV and 30 mA in the 2θ range of 10–110° with a step size of 0.02° and a counting time of 7 s/step, with LaB_6_ (SRM660b, NIST) as the internal standard. In addition, the fired samples were placed on Mo mesh and thinned by focused ion beam (FIB) milling (JEM-9320FIB, JEOL Ltd., Tokyo, Japan) with Ga ions to yield ~100 nm thick flakes; these flakes are half the average particle diameter of the raw ZnAl_2_O_4_ powder in size. For the samples thinned by FIB milling, transmission electron microscopy (TEM) at an accelerating voltage of 200 keV and energy-dispersive spectroscopy (EDS; JEM-2100, JEOL Ltd., Tokyo, Japan) were used to analyze the microstructures of the sintered samples and their elemental distributions.

## 3. Results and Discussion

### 3.1. Effect of the Cu_3_Nb_2_O_8_ Additive and Its Calcination on the Properties of ZnAl_2_O_4_

**Figure 2:**
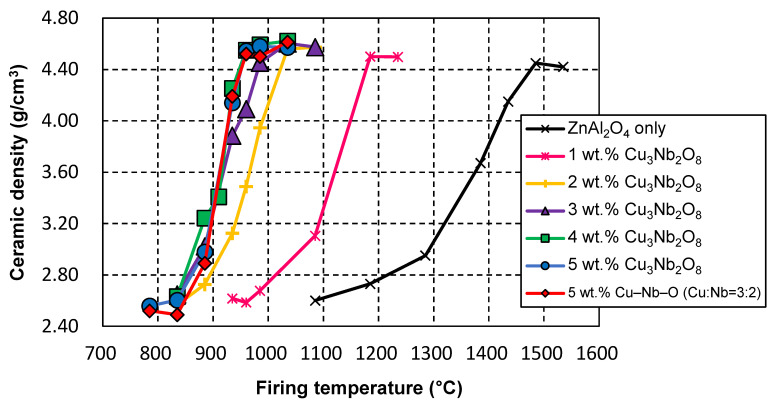
Ceramic densities of pristine ZnAl_2_O_4_, ZnAl_2_O_4_ containing 1, 2, 3, 4, and 5 wt.% of the Cu_3_Nb_2_O_8_ additive, and ZnAl_2_O_4_ containing 5 wt.% of the CuO–Nb_2_O_5_ (Cu:Nb = 3:2 (molar ratio)) additive as functions of firing temperature. The holding time at each temperature: 2 h.

We first evaluated the effect of the added Cu_3_Nb_2_O_8_ and the properties of the ZnAl_2_O_4_ sample. Figure 2 shows the relationship between the firing temperature and ceramic densities of ZnAl_2_O_4_ alone, and ZnAl_2_O_4_ containing 1, 2, 3, 4, and 5 wt.% of the Cu_3_Nb_2_O_8_ additive; samples were held for 2 h at each temperature. A firing temperature of 1485 °C was required for ZnAl_2_O_4_ to produce a dense sintered body in the absence of the sintering aid. On the other hand, significantly lower sintering temperatures were required for ZnAl_2_O_4_ containing the Cu_3_Nb_2_O_8_ additive; that is, dense sintered bodies with relative densities of 95% or more were obtained for samples containing 4 wt.% or more of the sintering aid when fired at 960 °C for 2 h. This temperature is only 1 °C lower than the melting point of metallic silver (961 °C); therefore, these bodies do not fully meet LTCC material requirements. However, they can be improved by prolonging the holding time at a lower firing temperature (see Section 3.2).

**Figure 3:**
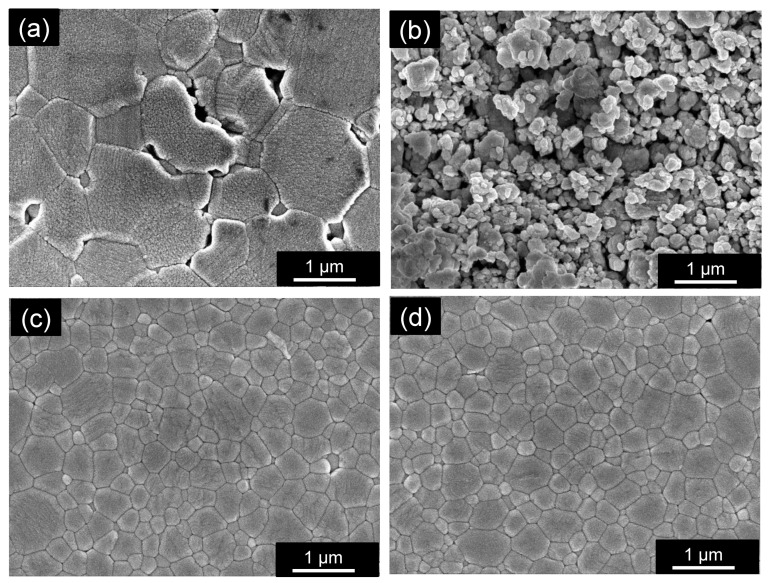
SEM images of polished surfaces of (**a**) pristine ZnAl_2_O_4_ fired at 1485 °C for 2 h (sample G01), (**b**) ZnAl_2_O_4_ containing 5 wt.% of the Cu_3_Nb_2_O_8_ additive fired at 785 °C for 2 h (G06), (**c**) ZnAl_2_O_4_ containing 5 wt.% of the Cu_3_Nb_2_O_8_ additive fired at 960 °C for 2 h (G07), and (**d**) ZnAl_2_O_4_ containing 5 wt.% of the CuO–Nb_2_O_5_ (Cu:Nb = 3:2 (molar ratio)) additive fired at 960 °C for 2 h (G08).

**Figure 4:**
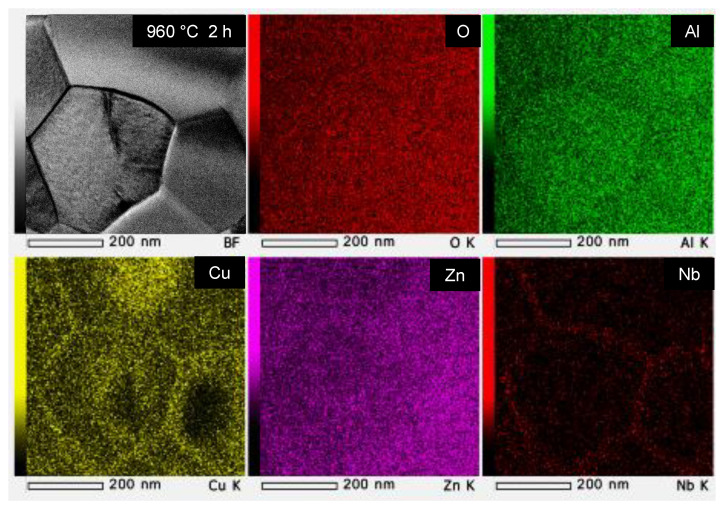
TEM image and EDS elemental maps of ZnAl_2_O_4_ containing 5 wt.% Cu_3_Nb_2_O_8_ fired at 960 °C for 2 h (G07).

Table 2 lists the additive quantities, additive conditions, firing temperatures, densities, average grain sizes (*D_g_*), dielectric properties (*ε_r_*, *Q* × *f*, *τ_f_*), and thermal conductivities (*κ*) of the main 14 samples (G01–14) examined in this study. For reference, typical data of a conventional LTCC (Al_2_O_3_ + glass) substrate are shown as sample R01. In addition, Figure 3 shows SEM images of polished surfaces that depict the microstructures of the sintered bodies. Figure 3a shows that the ZnAl_2_O_4_ sample fired at 1485 °C for 2 h (G01) contained grains approximately 1–2 μm in size (*D_g_*: 2.10 μm), which are larger than the grains of the raw powder (Figure 1b). However, submicron-sized voids were observed at various locations. Figure 3b,c show ZnAl_2_O_4_ sintered bodies containing 5 wt.% of the Cu_3_Nb_2_O_8_ additive. The sample fired at 785 °C for 2 h (G06) exhibited almost no necking between particles, with many voids present (Figure 3b). On the other hand, the sample fired at 960 °C for 2 h (G07) contained angular particles and few voids. The grains in Figure 3c (*D_g_*: 0.55 μm) are larger than those in Figure 3b (*D_g_*: 0.20 μm); however, they are still submicron in size. We conclude that the sample fired at 960 °C was densified. In addition, TEM–EDS (Figure 4) revealed that the Cu and Nb of the additive segregated at the grain boundaries; however, Cu was detected inside the ZnAl_2_O_4_ grains in some places.

As summarized in Table 2, samples G01–G05 and G07 were all well sintered, with relative densities in excess of 95%. Little change was observed in *ε_r_* with increasing amount of sintering aid. On the other hand, the *Q* × *f* of the ZnAl_2_O_4_ sample with 1 wt.% additive (G02, 98,200 GHz) was approximately sixfold higher than that of the additive-free sample (G01, 16,100 GHz), and decreased with further increases in additive quantity (G03–05 and G07). However, the *Q* × *f* value of sample G07 was observed to be 50,000 GHz or higher, even with 5 wt.% of the additive, which is a good value for low-temperature sintered ceramics. As has been extensively discussed, the main origins of the dielectric loss tangent (tan δ: reciprocal of the *Q* value) of an actual dielectric ceramic at microwave frequency is mainly determined by an intrinsic factor (the anharmonic terms of the crystal’s potential energy) and extrinsic factors (lattice defects caused by impurities, disordered charge distribution in the crystal, grain boundaries, random grain orientation, microcracks, porosity, etc.) [24,25,26]. Considering the above viewpoint, the relative density of sample G01 was 97.0%; hence, it was 3.0% porous. In addition, Figure 3a shows grains and void diameters that are sufficiently large to be recognizable in the SEM image. In other words, we believe that this sample contained many extrinsic dielectric loss factors associated with its porosity. The addition of 1 wt.% of the sintering aid decreased the number of voids in the sintered ZnAl_2_O_4_ (observed in Figure 3a), which reduced lattice relaxation and increased its *Q* × *f* value. Penn et al. reported that dielectric loss depends strongly on pore volume, with only a small degree of porosity markedly affecting the loss of sintered alumina [26]. They also mentioned that loss may be related to the surface area of the pores, as the alumina at the surface of a pore is in a different environment to the alumina within the matrix. The presence of a free surface (such as a pore) leads to crystal lattice relaxation. The relaxed surface is effectively different from that of the bulk material and is, therefore, likely to have a different tan δ (one of the extrinsic factors of dielectric loss). This study also supports our views on ZnAl_2_O_4_ (above), an Al-based oxide. Further increases in additive quantity probably resulted in higher degrees of lattice defects in the ZnAl_2_O_4_ due to Cu-component incorporation (see Section 3.3.2); hence, lower *Q* × *f* values were observed. In addition, the *Q* × *f* values of the samples were also affected by the relatively low *Q* × *f* of the Cu_3_Nb_2_O_8_ additive itself (G14, 18,700 GHz), which was presumably lower than that of ZnAl_2_O_4_ (106,000 GHz, reported by Zheng et al. [25]).

On the other hand, *τ_f_* was observed to increase slightly with increasing additive quantity compared with the value for the pristine ZnAl_2_O_4_ sample; that is, the sample devoid of additive (G01) exhibited a *τ_f_* value of −73 ppm·K^−1^, whereas sample G07, with 5 wt.% of the additive, exhibited a value of −56 ppm·K^−1^. The Cu_3_Nb_2_O_8_ additive itself (G14) had a *τ_f_* of −70 ppm·K^−1^, which is almost the same as that of the additive-free sample (G01); the additive quantity was also small. Therefore, we conclude that the cause of the observed trend rested with the base material rather than the additive and presume that the solid solution of the Cu component in ZnAl_2_O_4_ was involved. *κ* was also observed to decrease monotonically with increasing additive quantity. In particular, the *κ* value of the sample containing 1 wt.% of additive (G02) was dramatically lower (by a factor of two) compared to that of the additive-free sample (G01). On the basis of our previous research, we believe that the solid solution of the Cu component in ZnAl_2_O_4_ was responsible for this observation [17].

**Figure 5:**
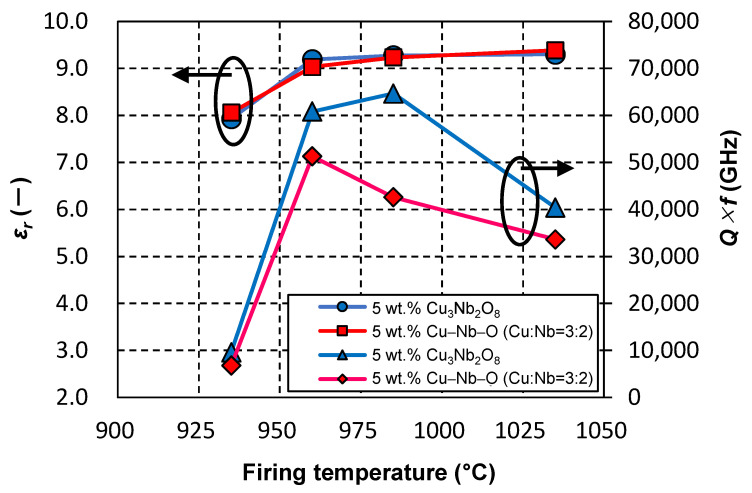
Effect of Cu–Nb–O additive (Cu:Nb = 3:2 (molar ratio)) calcination on the relationship linking firing temperature, relative permittivity, and the *Q* × *f* of ZnAl_2_O_4_ containing 5 wt.% of the additive. A 2 h holding time was applied at each temperature.

We next examined the ZnAl_2_O_4_ sample containing 5 wt.% of the CuO–Nb_2_O_5_ (Cu:Nb = 3:2 (molar ratio)) sintering aid, and compared various characteristics of its calcined and uncalcined samples. As shown in Figure 2, we did not detect any difference in the sintering behavior of the uncalcined (G08) and calcined (G07) samples. The SEM image in Figure 3d reveals that the microstructure of the uncalcined sample (G08) was almost identical to that of calcined G07 shown in Figure 3c; both exhibited almost no voids and submicron-sized grains (for G08, *D_g_* = 0.57 μm). The dielectric and thermal data summarized in Table 2 reveal no significant differences in the relative permittivities, *τ_f_* values, and thermal conductivities of G07 and G08; however, calcined sample G07 showed a higher *Q* × *f* value (60,800 GHz) than that of uncalcined sample G08 (51,300 GHz). Furthermore, Figure 5 shows that the two exhibited almost no difference in relative permittivity, whereas the calcined sample exhibited higher *Q* × *f* values at firing temperatures of 935, 960, 985, and 1035 °C.

**Figure 6:**
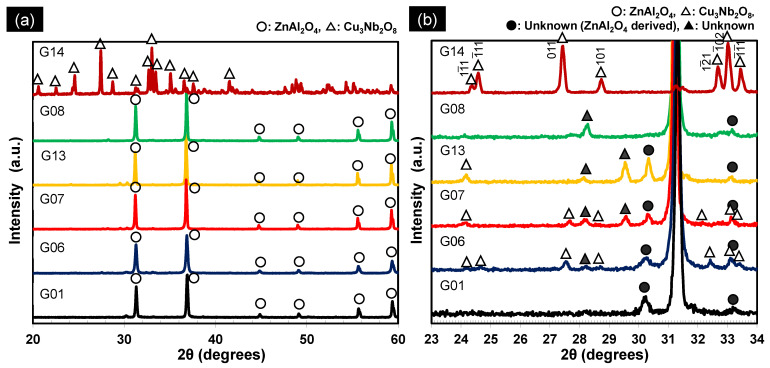
(**a**) XRD patterns of pristine ZnAl_2_O_4_ fired at 1485 °C for 2 h (G01), ZnAl_2_O_4_ containing 5 wt.% of the Cu_3_Nb_2_O_8_ additive fired at 785 °C for 2 h (G06), ZnAl_2_O_4_ containing 5 wt.% of the Cu_3_Nb_2_O_8_ additive fired at 960 °C for 2 h (G07), ZnAl_2_O_4_ containing 4 wt.% of the Cu_3_Nb_2_O_8_ additive fired at 960 °C for 384 h (G13), ZnAl_2_O_4_ containing 5 wt.% of the CuO–Nb_2_O_5_ (Cu:Nb = 3:2 (molar ratio)) additive fired at 960 °C for 2 h (G08), and pristine Cu_3_Nb_2_O_8_ fired at 885 °C for 2 h (G14). (**b**) Enlarged XRD patterns of G01, G06, G07, G13, G08, and G14 in panel (**a**). Cu_3_Nb_2_O_8_ diffraction indices are indicated in the figure.

The *Q* × *f* value of ZnAl_2_O_4_ appeared to depend on whether or not the CuO–Nb_2_O_5_ additive was calcined; hence, the reason for this dependence was investigated by XRD. Figure 6a shows XRD patterns of the G01, G06, G07, G13, G08, and G14 sintered bodies, with Figure 6b showing enlarged patterns. The unknown peaks indicated by solid circles (●) near 2θ = 30° and around 2θ = 33° were also observed in the pattern of the pure ZnAl_2_O_4_ sample (G01). Therefore, we conclude that these peaks are not directly related to the observed characteristic change. Peaks corresponding to Cu_3_Nb_2_O_8_ were observed for the calcined sample (G06) fired at 785 °C for 2 h, as well as for the sample (G07) fired at 960 °C for 2 h, although they were less intense in the case of the latter. In addition, unknown peaks, indicated by solid triangles (▲), were clearly observed. The XRD peaks observed for G06 and G07 were slightly shifted compared to those of the pure Cu_3_Nb_2_O_8_ sintered body (G14). For example, the 1$\bar{2}$1 reflection, which can be observed at 2θ = 32.6° in the XRD pattern of Cu_3_Nb_2_O_8_, was observed at lower angles in the patterns of G06 and G07. According to Kim et al. [19], such a shift is consistent with a solid solution in which Zn occupies Cu sites in Cu_3_Nb_2_O_8_. However, the same report revealed no significant change in the *Q* × *f* value due to the formation of the Zn solid solution. No peaks corresponding to Cu_3_Nb_2_O_8_ were observed for the sample with the uncalcined additive (G08) fired at 960 °C for 2 h. In addition, peaks corresponding to Zn_3_Nb_2_O_8_ were not observed. Cu_3_Nb_2_O_8_ and Zn_3_Nb_2_O_8_ reportedly exhibit relatively high *Q* × *f* values of approximately 50,000 and 80,000 GHz, respectively [19]. In other words, the formed Cu_3_Nb_2_O_8_ and Zn_3_Nb_2_O_8_ exhibit relatively high *Q* × *f* values when the sintered body is fabricated by the separate addition of sintering aids, such as CuO and Nb_2_O_5_. We, therefore, conclude that compounds related to CuO, Nb_2_O_5_, or ZnO, with lower *Q* × *f* values, were formed in the current work, which we presume to be the cause of the observed *Q* × *f* trend. Clearly, these results alone cannot identify the cause of the observed trend, and further detailed analysis is required.

### 3.2. Effect of Holding Time on the Properties of ZnAl_2_O_4_

**Figure 7:**
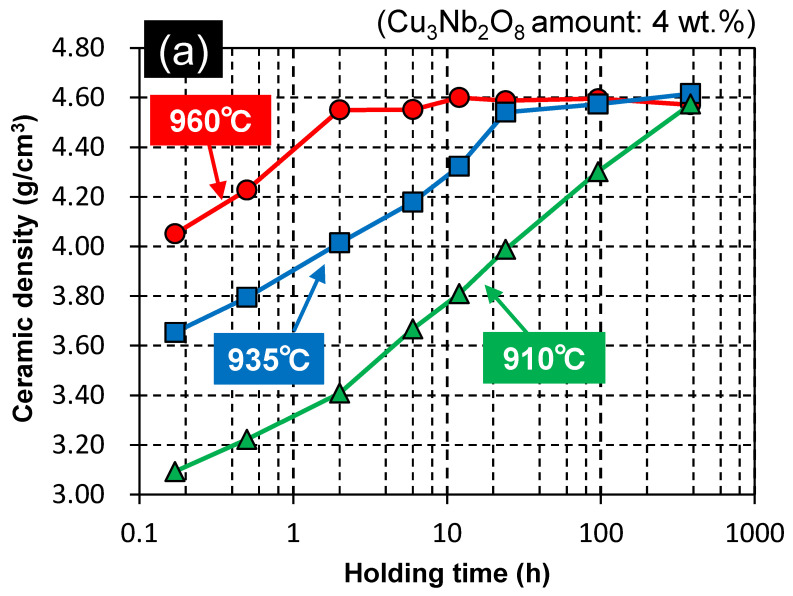

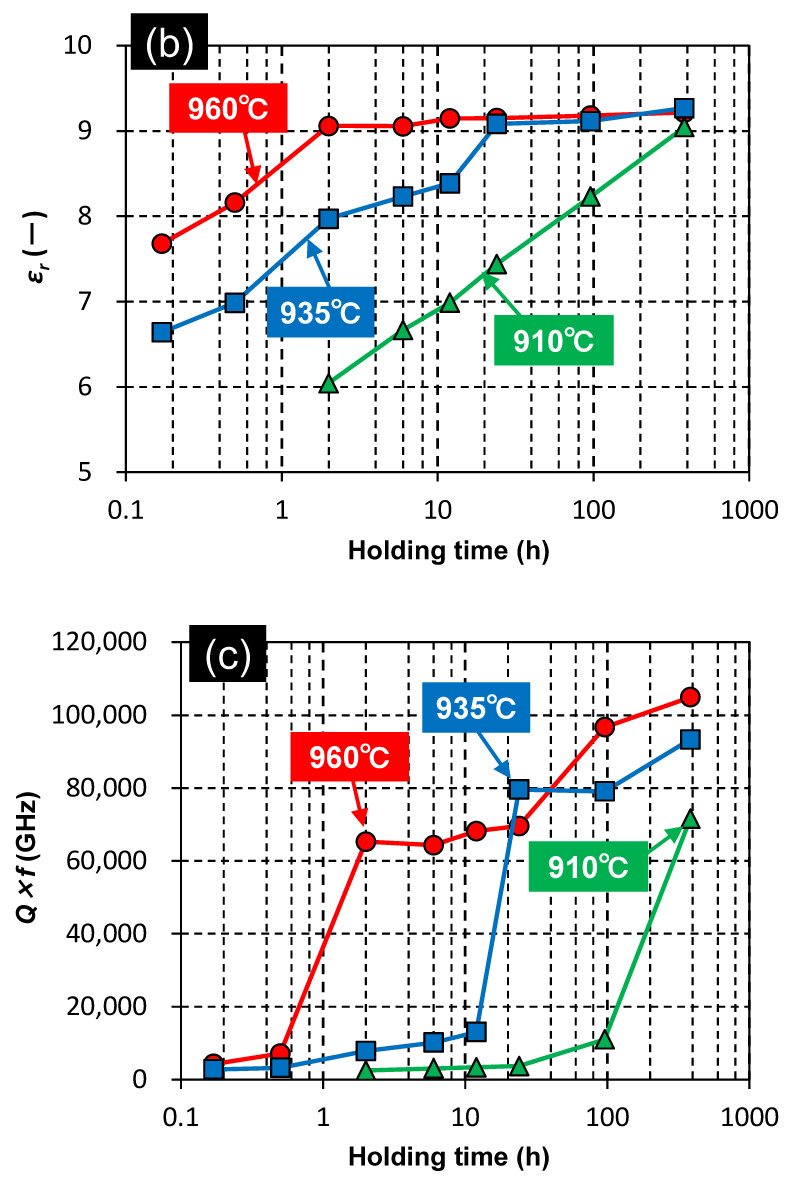
Ceramic densities and microwave dielectric and thermal properties of ZnAl_2_O_4_ containing 4 wt.% of Cu_3_Nb_2_O_8_ as a function of holding time at firing temperatures of 910, 935, and 960 °C: (**a**) ceramic density; (**b**) relative permittivity *ε_r_*; (**c**) *Q* × *f*.

Figure 2 shows that ZnAl_2_O_4_ sinterability saturated when more than 4 wt.% Cu_3_Nb_2_O_8_ was added. Therefore, the Cu_3_Nb_2_O_8_ quantity was fixed at 4 wt.% and the effects of retention time at 910, 935, and 960 °C, which are below the melting point of Ag, were investigated. As discussed in detail in the next section (Section 3.3), these temperatures were also lower than the melting temperature of the additive. Figure 7a reveals that the ceramic densities of the samples in this system were significantly affected by the time held at the firing temperature; that is, dense sintered bodies were obtained by holding for an extended time (384 h) even at firing temperatures less than or equal to 960 °C (e.g., 910 °C). Figure 7b,c show relative permittivity and *Q* × *f* data, while Table 2 summarizes sample properties (G05, G09–G13). Relative permittivity (Figure 7b) exhibited almost identical behavior to sintered body density (Figure 7a), with relative permittivity and density observed to saturate concurrently. On the other hand, the *Q* × *f* value (Figure 7c) rose sharply to a value of 60,000 GHz or higher (e.g., for G05 and G10) when the ceramic density of the sample exceeded a threshold value of 4.4–4.5 g/cm^3^. In addition, *Q* × *f* increased further with increasing holding time, even at the same density. The *Q* × *f* of the sample fired at 960 °C for 384 h (G13) was determined to be 105,000 GHz (at a frequency of ~13 GHz). This trend was also observed for samples fired for 384 h at lower temperatures (910 and 935 °C) (G10: 71,500, G12: 93,300 GHz). Moreover, the thermal conductivity of G13 exceeded 10 W·m^−1^·K^−1^ (10.1 W·m^−1^·K^−1^), which is approximately 10% higher than that of the sample fired at 960 °C for 2 h (G05). This trend was also observed for the samples fired at lower temperatures (910, and 935 °C) for 384 h (G10: 11.3, G12: 10.2 W·m^−1^·K^−1^); hence, G10, G12, and G13 are excellent LTCC candidate materials.

**Figure 8:**
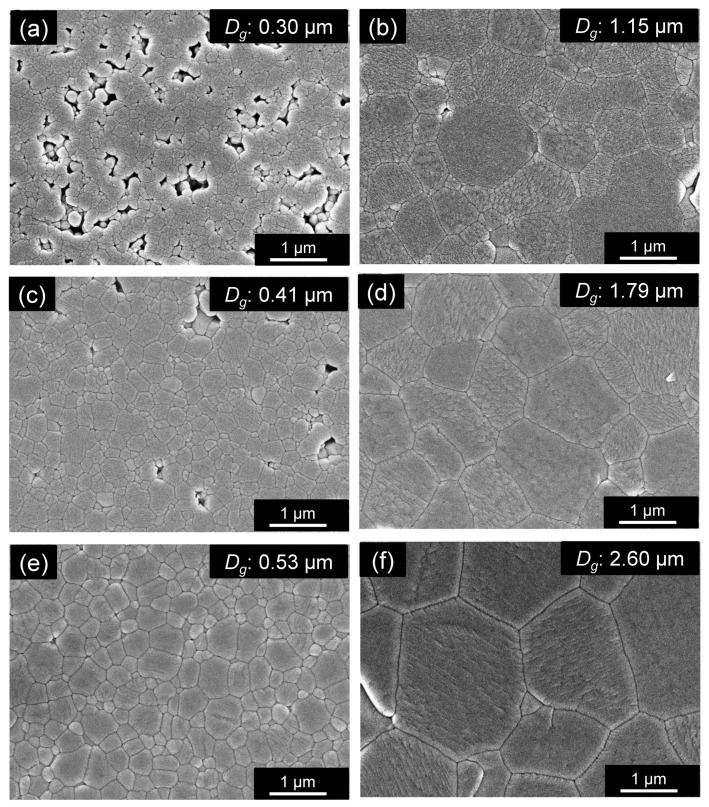
SEM images of polished surfaces of ZnAl_2_O_4_ containing 4 wt.% of the Cu_3_Nb_2_O_8_ additive fired at (**a**) 910 °C for 2 h (G09), (**b**) 910 °C for 384 h (G10), (**c**) 935 °C for 2 h (G11), (**d**) 935 °C for 384 h (G12), (**e**) 960 °C for 2 h (G05), and (**f**) 960 °C for 384 h (G13). The average grain size (*D_g_*) of each sample is also shown.

The reason for the observed increases in *Q* × *f* and thermal conductivity with prolonged holding time is discussed in terms of the SEM-observed sample microstructures. First, Figure 8a,c,e, reveal that all samples prepared with a retention time of 2 h (G09, G11, and G05) had submicron-sized grains, with higher firing temperatures leading to fewer voids. Almost no visible voids were observed in the sample fired at 960 °C for 2 h (G05). We presume that the porosity of the ZnAl_2_O_4_ sintered body suppresses crystal lattice relaxation (one of extrinsic factors of dielectric loss) [26]; hence, porosity is responsible for the higher *Q* × *f* of sample G05 compared with that of samples G09 and G11. Figure 8b,d,f reveal that the samples prepared with a retention time of 384 h (G10, G12, and G13) exhibited almost no voids. On the other hand, remarkable grain growth was observed, and many particles were larger than 1 μm; higher firing temperatures led to larger grains. Figure 8e,f compare the morphologies of the samples fired at 960 °C for 2 h (G05) and at 960 °C for 384 h (G13); hardly any voids and almost identical sintering densities were observed. On the other hand, sample G13 had the highest *Q* × *f* value in this study; it exhibited much higher grain growth (*D_g_*: 2.60 μm) than G05 (*D_g_*: 0.53 μm), with many particles larger than 2 μm. Therefore, larger grains may lead to fewer grain boundaries per unit volume (one of the extrinsic factors of dielectric loss) and higher *Q* × *f* values, which seem to have reduced the extrinsic dielectric loss factors and led to an equivalent *Q* × *f* value (106,000 GHz [25]) to that reported for pure ZnAl_2_O_4_. However, in the above study, the *Q* × *f* value was calculated to be 394,000 GHz when only the intrinsic factor was considered; hence, eliminating the influence of extrinsic factors remains an important issue. In addition, phonons have longer mean free paths that result in high heat conduction; we consider this to be related to the higher *Q* × *f* and thermal conductivity of the sample. For reference, the XRD pattern in Figure 6b shows that the phases generated in G13 (other than ZnAl_2_O_4_) at a retention time of 384 h were the same type as those observed for G08 (with a composition almost identical to that G05 with a retention time of 2 h), although their peak intensities were different. Identifying clear causes on the basis of these generated phases is a future objective.

### 3.3. Proposed Sintering Mechanism

#### 3.3.1. Melting Temperature

**Figure 9:**
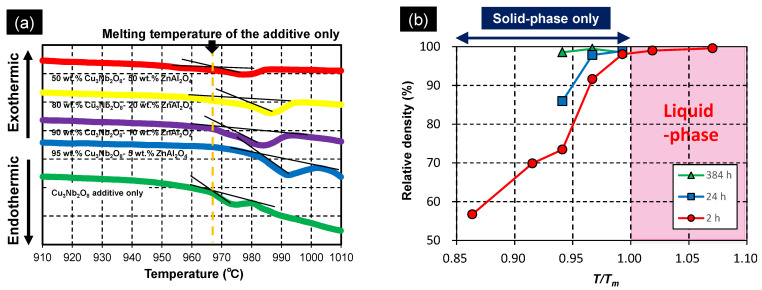
(**a**) Differential thermal analysis curves for Cu_3_Nb_2_O_8_/ZnAl_2_O_4_ mixtures acquired at 10 °C/min. (**b**) Relative densities of ZnAl_2_O_4_ containing 4 wt.% of Cu_3_Nb_2_O_8_ and held for 2, 24, and 384 h as a function of the firing-temperature/liquid-phase-formation temperature ratio (T/T_m_).

As discussed in Section 3.1, the ZnAl_2_O_4_ sample containing the Cu_3_Nb_2_O_8_ additive began to densify at a low temperature (885 °C, Figure 2). LTCC materials normally contain large quantities of glass, and densification is assumed to occur through liquid-phase sintering. Therefore, densification normally commences at a temperature above the melting point (eutectic temperature); hence, the melting temperatures of Cu_3_Nb_2_O_8_-additive/ZnAl_2_O_4_ mixtures need to be considered in addition to that of the Cu_3_Nb_2_O_8_ additive. Figure 9a presents DTA curves of 100:0, 95:5, 90:10, 80:20, and 50:50 (*w*/*w*) Cu_3_Nb_2_O_8_-additive/ZnAl_2_O_4_ mixtures when heated at 10 °C/min. The Cu_3_Nb_2_O_8_ additive melts at 967 °C, which did not change dramatically for most Cu_3_Nb_2_O_8_-additive/ZnAl_2_O_4_ powder mixtures. Moreover, this temperature is much higher than that at which densification begins (~885 °C, Figure 2), which suggests that densification occurs in the solid phase prior to liquid phase formation; this process was referred to as “solid-state-activated sintering” by German et al. [27,28]. The minimum melting temperature determined from the DTA curve of each sample was taken to be the liquid-phase formation temperature, T_m_ (K). Figure 9b depicts the relationship between T/T_m_ (where T (K) is the firing temperature) and the relative density of ZnAl_2_O_4_ containing 4 wt.% of Cu_3_Nb_2_O_8_ held for 2, 24, and 384 h. A T/T_m_ ratio above 1.00 indicates the formation of a liquid phase, while a value below 1.00 reveals that densification occurs solely through a solid-state mechanism. Samples at all retention times had relative densities of 98% or higher when T/T_m_ = 0.99 (T = 1233 K). Even with a T/T_m_ of 0.94 (T = 1183 K), the sample held for 384 h achieved a relative density of 98% or higher. Hence, ZnAl_2_O_4_ clearly underwent solid-state densification before liquification of the sintering aid; this system, therefore, underwent solid-state-activated sintering.

#### 3.3.2. Degree of Solid Solution

**Figure 10:**
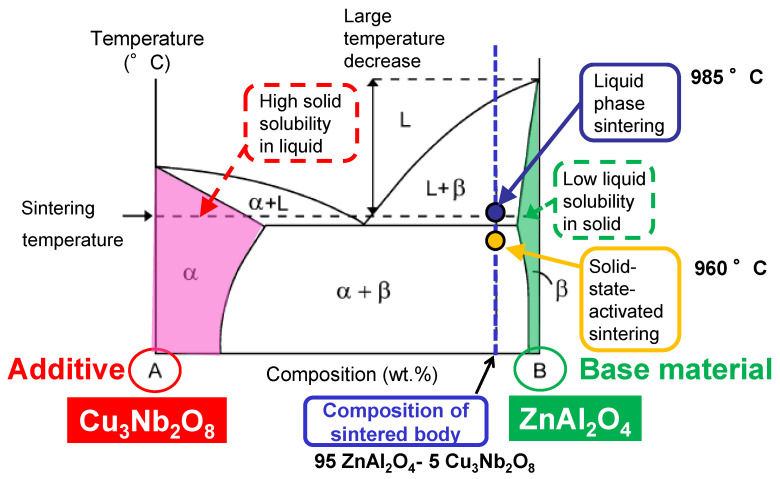
Ideal binary phase diagram for the liquid phase sintering of additive “A” and base material “B”.

German et al. [27,28] proposed “an ideal binary phase diagram for promoting liquid-phase sintering (LPS)’’ (Figure 10). The composition and firing temperature of the actual liquid-phase sintered body correspond to the blue dot in Figure 10. An ideal combination of composition and temperature results in high solid solubility in the liquid (eutectic liquid in this diagram) and low solubility of the liquid in the solid. A significantly lower melting temperature compared to that of the single component affords a processing temperature advantage. Therefore, we conclude that the conditions for promoting liquid-phase sintering is satisfied in our case, with ZnAl_2_O_4_, as the base material, exhibiting higher solid solubility in the Cu_3_Nb_2_O_8_ additive than that of the Cu_3_Nb_2_O_8_ additive in ZnAl_2_O_4_.

Although the proposed theory (above) is only intended to describe the behavior of samples when liquid phases form, we believe that the described behavior also occurred in the solid state in this study (solid-state-activated sintering; yellow dot in Figure 10). However, as the phase diagram for this system has not yet been reported, we assumed a pseudo-binary phase diagram for the ZnAl_2_O_4_–Cu_3_Nb_2_O_8_ system. Accordingly, we determined the degrees of solid dissolution of the Cu_3_Nb_2_O_8_ additive in ZnAl_2_O_4_, and ZnAl_2_O_4_ in the Cu_3_Nb_2_O_8_ additive by monitoring lattice constant changes. Specifically, two types of mixed powder sample were heat-treated: 95 wt.% Cu_3_Nb_2_O_8_ additive + 5 wt.% ZnAl_2_O_4_ (95C05Z), and 95 wt.% Cu_3_Nb_2_O_8_ additive + 5 wt.% ZnAl_2_O_4_ (95Z05C). Changes in the XRD-measured lattice constants (unit cell volumes in this case) were determined as a function of heat-treatment temperature. The lattice constant of Cu_3_Nb_2_O_8_ after calcination at 835 °C for 2 h was measured for the 95C05Z sample, while the lattice constant of ZnAl_2_O_4_ after calcination at 1100 °C for 4 h was measured for the 95Z05C sample, with values prior to heat treatment used as reference values for calculating change ratios.

**Figure 11:**
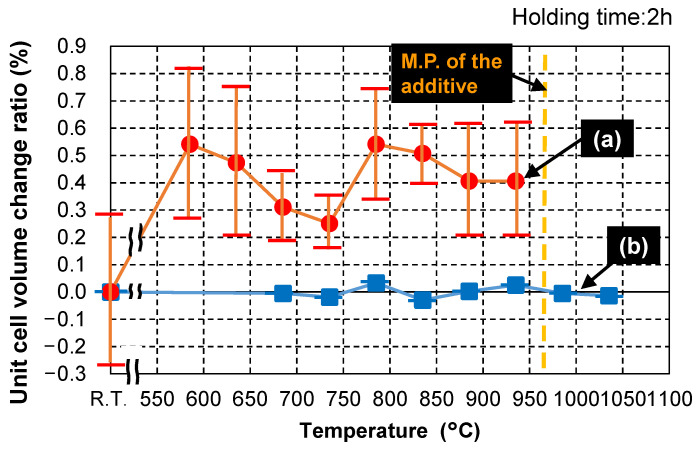
Unit cell volume change ratios of (**a**) Cu_3_Nb_2_O_8_ in a Cu_3_Nb_2_O_8_ sample containing 5 wt.% of ZnAl_2_O_4_ (95C05Z), and (**b**) ZnAl_2_O_4_ in a ZnAl_2_O_4_ sample containing 5 wt.% of the Cu_3_Nb_2_O_8_ additive (95Z05C) as a function of heat-treatment temperature determined by XRD. Each particular temperature was held for 2 h.

Figure 11 displays the relationship between heat-treatment temperature and unit cell volume change ratios; the error bars show standard deviations (σ). Figure 11a reveals that the Cu_3_Nb_2_O_8_ unit cell volume change ratio was approximately 0.25–0.55% higher than the reference value (at room temperature, 20 °C) when heat-treated at 585 °C or higher, although these results had relatively large error bars. This suggests that Zn^2+^, which has a larger ionic radius (0.074 nm) than Cu^2+^ (0.073 nm) and Nb^5+^ (0.064 nm) when six-coordinated [29], was incorporated into Cu_3_Nb_2_O_8_. It is not clear whether or not Al^3+^, which has a smaller ionic radius (0.0535 nm when six-coordinated) than Cu^2+^ or Nb^5+^, was incorporated in Cu_3_Nb_2_O_8_. However, Al^3+^ was possibly an interstitial solid solution; in this case, a lower unit cell volume would not be expected for Cu_3_Nb_2_O_8_.

On the other hand, Figure 11b shows almost no change in ZnAl_2_O_4_ unit cell volume ratio (less than 0.05%) when heat-treated at 685–1035 °C, which suggests that Cu^2+^, with a smaller ionic radius than Zn^2+^, but larger than Al^3+^ [29], was not significantly incorporated into the Zn and Al sites of ZnAl_2_O_4_. Alternatively, it is possible that Cu^2+^ was simultaneously incorporated into both the Zn sites of ZnAl_2_O_4_ and the Al sites of ZnAl_2_O_4_, which would offset any increase/decrease in the ZnAl_2_O_4_ unit cell volume. Shigeno et al. [9] reporteded a unit cell volume change ratio of only ~0.1% for Al_2_O_3_ when Cu^2+^ and Ti^4+^ were incorporated into Al_2_O_3_ in an alumina sample containing 5 wt.% of the Cu–Ti–Nb–O sintering aid. In addition, Phillips et al. [30] reported that the unit cell volume of Ti-doped sapphire was also only ~0.1% larger than that of pure sapphire after annealing at 1600 °C for 24 h, which is similar to the results obtained in this study. Therefore, even if Cu^2+^ was incorporated into the Al sites of ZnAl_2_O_4_, we estimate that the unit cell volume change ratio (increase ratio) was only about 0.1%, while the change ratio (decrease ratio) in the unit cell volume associated with incorporating Cu^2+^ into the Zn sites was estimated to be ~0.1%. In other words, the ZnAl_2_O_4_ unit cell volume change ratio (Figure 11b) was estimated to be smaller than that of Cu_3_Nb_2_O_8_ (Figure 11a) (0.25–0.55%).

**Figure 12:**
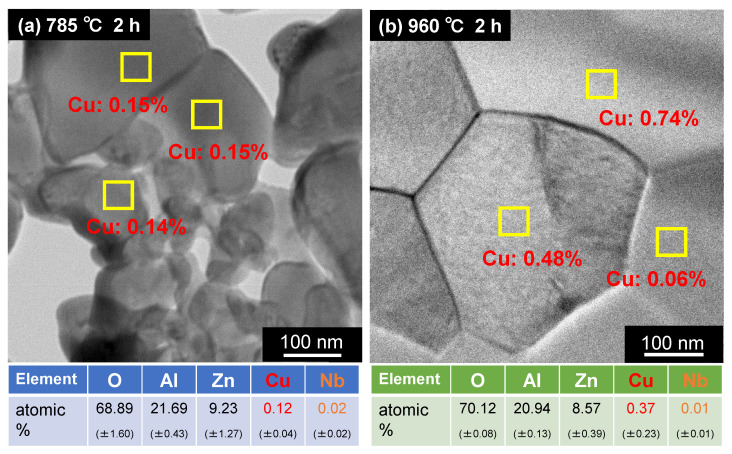
TEM images and EDS data (including standard deviation: σ) for intragranular atoms (O, Al, Zn, Cu, and Nb) in sintered ZnAl_2_O_4_ containing 5 wt.% of Cu_3_Nb_2_O_8_ fired at (**a**) 785 °C for 2 h (G06), and (**b**) 960 °C for 2 h (G07).

To gain further insight into the Cu and Nb distributions, the ZnAl_2_O_4_ particles were subjected to TEM–EDS before and after densification. Figure 12 shows TEM images and continuous EDS data for the intragranular additive elements (Cu and Nb) in sintered ZnAl_2_O_4_ containing 5 wt.% of Cu_3_Nb_2_O_8_ fired at different temperatures (785 °C for 2 h, and 960 °C for 2 h). Each sample was subjected to six 30 min continuous EDS analyses at different intragranular locations; the values shown in parentheses denote standard deviations. Approximately 0.12 ± 0.04 at.% Cu was detected in the ZnAl_2_O_4_ sample (G06) fired at 785 °C for 2 h (Figure 12a). However, as shown in Figure 2a, this sample was not densified and is, therefore, considered to be a solid solution of Cu with the same valence as Zn (i.e., divalent). On the other hand, approximately 0.37 ± 0.23 at.% Cu was detected in the densified ZnAl_2_O_4_ sample (G07) fired at 960 °C for 2 h (Figure 12b). As evidenced by the standard deviations in Figure 12b and the EDS elemental map shown in Figure 4, Cu was not uniform and was sparsely distributed within the ZnAl_2_O_4_ grain. The reason for this sparse distribution remains unknown; however, we presume that one of the reasons is that the Cu diffuses slowly during solid-state sintering, unlike in liquid-phase sintering. Nonetheless, we can confirm that more Cu diffused into the densified ZnAl_2_O_4_ grains than in grains before densification, suggesting that Cu was incorporated into ZnAl_2_O_4_ not only at Zn sites but also at some Al sites. In contrast, the amounts of Nb were determined to be much less than 0.1 at.%, which is regarded to be the detection limit of the EDS instrument used. No clear difference in this value was observed before and after densification; therefore, while we cannot provide a definitive conclusion because only local analyses were performed, we presume that Nb atoms were not present within the ZnAl_2_O_4_ particle grains.

The abovementioned results reveal that ZnAl_2_O_4_ mainly densified with the help of defects formed by the substitution of Cu^2+^ for Al^3+^ in the ZnAl_2_O_4_ lattice in the solid state at 835–960 °C, and they are believed to have been generated by the following reactions (Kröger–Vink notation):

(3) $$2CuO\vec{Al_{2}O_{3}}2Cu_{Al}^{\prime }+V_{O}^{˙˙}+2O_{O}^{\times },$$

and

(4) $$6CuO+Al_{2}O_{3}\vec{Al_{2}O_{3}}6Cu_{Al}^{\prime }+9O_{O}^{\times }+2Al_{i}^{˙˙˙}.$$

Reaction (3) assumes defects due to vacancy formation, while Reaction (4) assumes defects due to interstitial ion formation. In the present study, oxygen vacancies ($V_{O}^{˙˙}$ in Reaction (3)) or interstitial Al^3+^ ($Al_{i}^{˙˙˙}$ in Reaction (4)) were the main defects, as indicated by the significantly higher Cu concentration in the densified ZnAl_2_O_4_ grains. Nb atoms are assumed to assist in increasing the diffusion rate of Cu^2+^ by lowering the melting temperature of the sintering additive. The addition of Nb_2_O_5_ to CuO lowered the melting temperature from 1025 to 967 °C. Furthermore, although the ionic radius of Nb^5+^ is relatively close to that of Al^3+^, they cannot substitute into Al^3+^ sites due to the +2 difference in valence.

The abovementioned results suggest that our system is qualitatively consistent with the “ideal binary phase diagram of LPS” advocated by German et al. [27,28]; therefore, they support the solid-state-activated sintering mechanism.

#### 3.3.3. Solid-State-Activated Sintering Model in This Study

**Figure 13:**
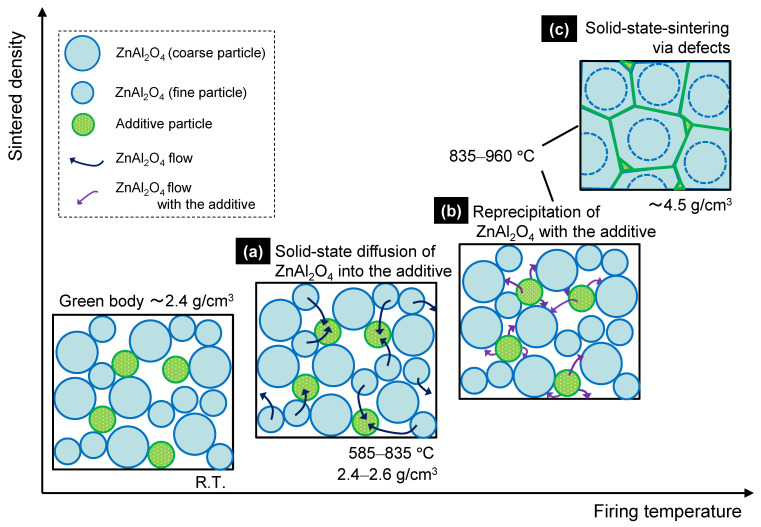
Solid-state-activated sintering model for ZnAl_2_O_4_ containing 5 wt.% of Cu_3_Nb_2_O_8_. (**a**) Solid-state diffusion of ZnAl_2_O_4_ into the additive, (**b**) Reprecipitation of ZnAl_2_O_4_ with the additive, and (**c**) Solid-state-sintering via defects.

A schematic of the solid-state-activated sintering mechanism in this study is shown in Figure 13. Fine ZnAl_2_O_4_ particles are gradually incorporated into the additive at temperatures below 585 °C, without considerable densification (Figure 13a), and a solid solution forms at temperatures up to 835 °C, at which point saturation occurs and ZnAl_2_O_4_ reprecipitates. According to the Kelvin equation [31], reprecipitation occurs preferentially on the coarse particles, which are less soluble than the fine particles (Figure 13b) through a process known as “Ostwald ripening.” Necks form between coarse particles as they grow. The neck region is less soluble than the coarse particles; hence, reprecipitation occurs preferentially at the necks. TEM–EDS (Figure 12) revealed that the deposited ZnAl_2_O_4_ was not pure, and those small quantities of additive components (Cu^2+^) were incorporated into the Zn^2+^ sites, as well as the Al^3+^ sites of the ZnAl_2_O_4_ in neck regions. We presume that the incorporated atoms created defects (Al- or O-related defects) in the ZnAl_2_O_4_ lattice that promote solid-phase diffusion between ZnAl_2_O_4_ particles, thereby promoting sintering (Figure 13c). The solid solution particles of the additive probably formed an intergranular amorphous phase during densification [32], which reduced the grain-boundary energy of ZnAl_2_O_4_. As sintering occurs through solid-phase diffusion, long retention times are required; however, densification is guaranteed. Lastly, according to the TEM–EDS results (Figure 4), we conclude that most additive components segregated and remained in the intergranular amorphous phase and other crystal compounds at grain boundaries. Because the ZnAl_2_O_4_ grains were larger after sintering for 384 h than after sintering for 2 h (Figure 8b,d,f), we conclude that the grains increased in size during sintering.

## 4. Conclusions

The addition of a small amount of the calcined Cu_3_Nb_2_O_8_ sintering aid to ZnAl_2_O_4_, a highly thermally conductive dielectric material, successfully enabled ZnAl_2_O_4_ sintering at (low) temperatures below the melting point of silver (961 °C), resulting in better dielectric properties (in particular, a quality factor by resonant frequency product *Q* × *f*) than those obtained by the addition of the non-calcined CuO–Nb_2_O_5_ (in a 3:2 Cu:Nb molar ratio) sintering aid. In addition, the sample fired at 960 °C exhibited more suitable dielectric characteristics, such as a relative permittivity *ε_r_* of 9.2, a *Q* × *f* of 105,000 GHz, and a temperature coefficient of resonant frequency *τ_f_* of −56 ppm·K^−1^ when the holding time was prolonged from 2 h to 384 h. The sample exhibited a thermal conductivity of 10.1 W·m^−1^·K^−1^, which exceeds that of conventional LTCCs (~2–7 W·m^−1^·K^−1^). Moreover, we suggest that this system underwent solid-state-activated sintering by specifically incorporating Cu^2+^ in the ZnAl_2_O_4_ lattice, in which densification was virtually complete even when the sintering aid remained in the solid state. The Nb component presumably indirectly affected the Cu^2+^ diffusion rate by lowering the melting temperature of the additive.

Future studies will further elucidate the mechanism associated with solid-state-activated sintering in this system and apply the acquired knowledge to the fabrication of various Al-based ceramic materials.

## Figures and Tables

**Table 1:** ZnAl_2_O_4_ and additive quantities, additive conditions, firing temperatures, holding times, and comparison purposes of the main 14 samples examined in this study. Comparison purposes: *1) clarifying the effect of additive quantities for well-sintered samples; *2) clarifying the effect of firing temperature for samples of the same composition (ZnAl_2_O_4_ with 5 wt.% of calcined additive); *3) clarifying the effect of additive calcination; *4) clarifying the effect of prolonging holding times for samples fired at 910, 935, and 960 °C (retention times: 2 and 384 h).

Sample	ZnAl_2_O_4_ Quantity	Additive Quantity	Additive Conditions	Firing Temperature	Holding Time	Comparison Purpose
		wt.%		°C	h	
G01	100	0	Calcined	1485	2	*1)
G02	99	1	Calcined	1185	2	*1)
G03	98	2	Calcined	1085	2	*1)
G04	97	3	Calcined	1035	2	*1)
G05	96	4	Calcined	960	2	*1) *4)
G06	95	5	Calcined	785	2	*2)
G07	95	5	Calcined	960	2	*1) *2) *3)
G08	95	5	Non-calcined	960	2	*3)
G09	96	4	Calcined	910	2	*4)
G10	96	4	Calcined	910	384	*4)
G11	96	4	Calcined	935	2	*4)
G12	96	4	Calcined	935	384	*4)
G13	96	4	Calcined	960	384	*4)
G14	0	100	Calcined	885	2	*1)

**Table 2:** Additive quantities and conditions, firing temperatures, densities, average grain sizes (*D_g_*), dielectric properties (*ε_r_*, *Q* × *f*, *τ_f_*), and thermal conductivities (*κ*) of the samples examined in this study; “✕” indicates that the values of ε_r_ and *Q* × *f* of the sample (G06) were unable to be determined due to their significantly low values; “✕✕” indicates that the values ofτ_f_ and κ for the samples (G06, G09, and G11) with insufficient ceramic densities (relative densities less than 95%) were not measured. R01* is an example of a conventional LTCC substrate.

Sample	Additive Quantity	Additive Conditions	Firing Temp.	Holding Time	Ceramic Density	Relative Density	*D_g_*	*ε_r_*	*Q* × *f*	*τ_f_*	*κ*
	wt.%		°C	h	g/cm^3^	%	μm	—	GHz	ppm·K^−1^	W·m^−1^·K^−1^
G01	0	Calcined	1485	2	4.47	97.0	2.10	9.0	16,100	−73	27.3
G02	1	Calcined	1185	2	4.50	97.5	3.10	9.0	98,200	−63	12.2
G03	2	Calcined	1085	2	4.57	98.9	1.36	9.1	81,700	−60	10.2
G04	3	Calcined	1035	2	4.60	99.4	0.89	9.3	77,000	−60	8.8
G05	4	Calcined	960	2	4.55	98.1	0.53	9.1	65,300	−54	8.9
G06	5	Calcined	785	2	2.56	55.0	0.20	✕	✕	✕✕	✕✕
G07	5	Calcined	960	2	4.55	97.8	0.55	9.2	60,800	−56	7.9
G08	5	Non-calcined	960	2	4.52	97.2	0.57	9.0	51,300	−63	8.5
G09	4	Calcined	910	2	3.41	73.5	0.30	6.0	2480	××	××
G10	4	Calcined	910	384	4.57	98.6	1.15	9.0	71,500	−54	11.3
G11	4	Calcined	935	2	4.01	86.4	0.41	8.0	7840	××	××
G12	4	Calcined	935	384	4.62	99.5	1.79	9.3	93,300	−61	10.2
G13	4	Calcined	960	384	4.57	98.5	2.60	9.2	105,000	−56	10.1
G14	100	Calcined	885	2	5.37	95.0	10.1	15.1	18,700	−70	6.1
R01*	—	—	900–1100 [4]	—	—	—	—	<15 [4,5]	>1000 [4]	<±100 [4]	2–7 [4,5]

## Data Availability

Data will be made available upon reasonable request.
